# Preclinical test of a lentivirus-mediated RNAi gene therapy against HIV-AIDS in the humanized mouse model

**DOI:** 10.1186/1742-4690-8-S2-P9

**Published:** 2011-10-03

**Authors:** Mireille Centlivre, Nicolas Legrand, Ying-Poi Liu, Karin J von Eíje, Kees Weijer, Bianca Blom, Hergen Spits, Ben Berkhout

**Affiliations:** 1 Laboratory of Experimental Virology, Department of Medical Microbiology, Center for Infection and Immunity Amsterdam (CINIMA), Academic Medical Center of the University of Amsterdam (AMC-UvA), Amsterdam, The Netherlands; 2 Department of Cell Biology & Histology, Center for Immunology of Amsterdam (CIA), AMC-UvA, Amsterdam, The Netherlands; 3 AIMM Therapeutics, AMC-UvA, Amsterdam, The Netherlands

## Background

HIV-1 is still a major public health problem and one of the priorities of the World Health Organization. The development of HAART against HIV was a considerable advance for infected individuals, but this life-long treatment does only block virus replication, and no viral eradication is obtained. Furthermore, HAART may exhibit long-term toxicity and may eventually lead to the emergence of drug-resistant viral variants. We explore a new durable therapeutic intervention based on a gene therapy that induces RNA interference (RNAi) against HIV-1. In this pre-clinical research setting, "humanized" experimental mouse models are of interest considering the relative ease of handling and relatively low cost as compared to non-human primates.

## Methods

We have developed an RNAi gene therapy based on the transduction of human hematopoietic progenitor cells (HPC) with lentiviral vectors encoding short-hairpin RNAs to induce silencing of HIV genes. We have tested the efficacy and safety of such a shRNA-based gene therapy strategy in the "Human Immune System" (HIS) BALB/c Rag2^-/-^IL-2Rγ_c_^-/-^ mouse model, which are reconstituted with human HPC that were first transduced *ex vivo* with a lentiviral vector expressing the antiviral shRNAs.

## Results

We observed a normal *in vivo* development of the human immune system with a good recovery of human shRNA+cells for the candidate shPol47, shPol1 and shRT5 inhibitors. However, the *in vivo* recovery of human shGag5-transduced cells was extremely poor, suggesting a negative impact of this specific shRNA on the development of the human immune system. When these 4 shRNAs were combined in a single lentiviral vector (R4), we observed a similar negative off-target effect due to the shGag5 component. Upon removal of shGag5 as in vector R3, transduction of human HPC results in a normal differentiation of the human immune system, highlighting the *in vivo* safety of this candidate R3 gene therapy vector for a clinical trial. Moreover, human HPC expressing the antiviral shNef generate human CD4+T cells with the ability to resist HIV-1 replication in a sequence specific manner.

## Conclusion

Overall, these results underscore the usefulness of the HIS (BALB-Rag/γ) mouse model for testing the safety and efficacy of durable anti-HIV gene therapy approaches. In this model, human HPC expressing anti-HIV-1 shRNA give rise to multi-lineage reconstitution of the immune system *in vivo* and generate CD4^+^ T cells that are not susceptible for HIV-1 replication.

